# Metabolite profiling in posttraumatic stress disorder

**DOI:** 10.1186/s40303-015-0007-3

**Published:** 2015-02-08

**Authors:** Alexander Karabatsiakis, Gilava Hamuni, Sarah Wilker, Stephan Kolassa, Durairaj Renu, Suzanne Kadereit, Maggie Schauer, Thomas Hennessy, Iris-Tatjana Kolassa

**Affiliations:** Clinical & Biological Psychology, Ulm University, Albert-Einstein Allee 47, 89081 Ulm, Germany; SAP Switzerland AG, Tägerwilen, Switzerland; Strand Life Sciences Pvt. Ltd., Bangalore, India; Department of Biology, University of Konstanz, Konstanz, Germany; Clinical Psychology & Neuropsychology, University of Konstanz, Konstanz, Germany; Agilent Incorporated, Singapore, Republic of Singapore

**Keywords:** Posttraumatic stress disorder, Metabolite profiling, Mass spectrometry, Biological pathways, Palmitoylethanolamide, Glycerophospholipid

## Abstract

**Background:**

Traumatic stress does not only increase the risk for posttraumatic stress disorder (PTSD), but is also associated with adverse secondary physical health outcomes. Despite increasing efforts, we only begin to understand the underlying biomolecular processes. The hypothesis-free assessment of a wide range of metabolites (termed metabolite profiling) might contribute to the discovery of biological pathways underlying PTSD.

**Methods:**

Here, we present the results of the first metabolite profiling study in PTSD, which investigated peripheral blood serum samples of 20 PTSD patients and 18 controls. We performed liquid chromatography (LC) coupled to Quadrupole/Time-Of-Flight (QTOF) mass spectrometry. Two complementary statistical approaches were used to identify metabolites associated with PTSD status including univariate analyses and *Partial Least Squares Discriminant Analysis* (PLS-DA).

**Results:**

Thirteen metabolites displayed significant changes in PTSD, including four glycerophospholipids, and one metabolite involved in endocannabinoid signaling. A biomarker panel of 19 metabolites classifies PTSD with 85% accuracy, while classification accuracy from the glycerophospholipid with the highest differentiating ability already reached 82%.

**Conclusions:**

This study illustrates the feasibility and utility of metabolite profiling for PTSD and suggests lipid-derived and endocannabinoid signaling as potential biological pathways involved in trauma-associated pathophysiology.

**Electronic supplementary material:**

The online version of this article (doi:10.1186/s40303-015-0007-3) contains supplementary material, which is available to authorized users.

## Background

Traumatic events, such as war, torture, rape or natural disasters, cumulatively increase the risk of developing posttraumatic stress disorder (PTSD) [1]. According to the Diagnostic and Statistical Manual of Mental Disorders (DSM), this mental health disorder is defined by the joint presence of four symptom clusters: intrusive re-experiencing of the traumatic event(s), avoidance of trauma-reminders, alterations in mood and cognition, and hyperarousal [2]. In addition to suffering from distressing traumatic memories, survivors with PTSD are also at enhanced risk of adverse physical health outcomes, including cardiovascular diseases and auto-immune diseases [3]. In recent years, increasing effort has been devoted to unraveling the underlying biomolecular ‘memories’ of traumatic stress in order to better understand both the disorder etiology as well as the observed co-morbidity with physical diseases. PTSD-associated alterations have been reported in the neuroendocrine system [4,5] and the immune system [6-8]. Furthermore, several lines of evidence point toward accelerated age-related processes in PTSD, reflected for instance in shortened telomere length [9], enhanced DNA damage [10] or an altered N-glycosylation profile [11]. These studies have largely contributed to our current understanding of the cellular and molecular alterations in PTSD, but are nevertheless confined to the investigation of biological pathways already known or hypothesized to be involved in PTSD etiology, symptomatology and associated health impairments.

However, the recent development of high-throughput technology enables the untargeted investigation of thousands of biological markers, and hence opens new opportunities for the discovery of so far unknown biomolecular pathways of disorders and diseases. These newly developed ‘omics’ approaches comprise the global study of DNA (‘genomics’), gene expression (‘transcriptomics’), protein expression (‘proteomics’), and lipids (‘lipidomics’). One of the latest omic sciences, metabolomics, employs mass spectrometry to investigate the metabolome, defined as the collection of small molecules (metabolites) which can be found in a given biological sample (e.g., cells, tissue or extracellular liquid) [12-14]. The metabolome represents the final outcome of environmentally influenced gene regulation and protein expression and thus “serves as a direct signature of biochemical activity” ([15], p.263). Therefore, it exhibits the strongest link with the overall health status of an individual [12].

So far, only a limited number of studies employed this new method to investigate metabolite profiles in psychiatric disorders such as depression [16-19] and schizophrenia [20-23]. These studies illustrate the large potential of metabolomics to contribute to a deeper understanding of the pathophysiological alterations associated with mental health disorders and to identify novel biomarker candidates and their associated pathways. For instance, metabolite profiling studies of depression in both urine and plasma identified several metabolites involved in disturbed energy metabolism to be altered in depression, a finding that corresponds well with the commonly observed psychosomatic symptoms of fatigue and lethargy in depressed individuals [18,19]. The clinical relevance of metabolite profiling has been further demonstrated by an investigation which gave initial evidence that responders and non-responders to pharmacological treatment for depression could be differentiated by this approach [16].

To our best knowledge, this is the first study employing metabolite profiling to identify so far unknown metabolites associated with a diagnosis of PTSD. We hypothesized that we would be able to identify a profile of altered metabolite levels associated with the diagnosis of PTSD in the aftermath of traumatic stress. A biomarker panel of 19 identified metabolites enabled the relatively precise classification of the PTSD status. Furthermore, this novel methodological approach identified potential novel biological pathways implied in PTSD etiology including lipid-derived signaling.

## Methods

### Subjects

We investigated the metabolite profile in peripheral blood serum of 20 trauma-exposed individuals with a diagnosis of PTSD according to DSM-IV-TR [24] and 18 healthy controls with varying degrees of trauma exposure. All participants were recruited at the Center of Excellence for Psychotraumatology, University of Konstanz, Germany, and via public advertisement. Participants were included in the present study if they met the following criteria (1) age between 18 and 55, (2) no psychotropic medication, (3) no autoimmune disease, (4) no signs of a current infection according to the whole blood count, and (5) no substance addiction. PTSD cases and controls were matched based on age and ethnicity. For an overview on demographics and clinical variables of the groups see Table 1.

**Table 1:** **Demographic and clinical sample characteristics**

	**Controls (N = 18)**	**PTSD (N = 20)**	**Statistic** ^**b**^	***p*** **-value**
N Female (%)	8 (44)	11 (55)	Fisher’s exact test	.75
Mean Age in Years (SD)	31.06 (10.93)	32.65 (8.46)	*t*_df=36_ = −.51	.62
N Smoker (%)	11 (61)	12 (60)	Fisher’s exact test	1.00
Mean Cigarettes per Day (SD)	5.58 (9.74)	7.72 (13.24)	W = 172.50	.82
Region of Origin			Fisher’s exact test	.49
N Africa (%)	7 (39)	5 (25)		
N Middle East (%)	11(61)	14 (70)		
N Southeastern Europe (%)	0 (0)	1 (5)		
N Insecure Asylum Status (%)^a^	7 (39)	20 (100)	Fisher’s exact test	< .001
Mean Number of CAPS Events (SD)	5.56 (2.94)	8.35 (1.95)	*t*_df=36_ = −3.50	.001
Mean CAPS Score (SD)	9.11 (15.41)	84.5 (18.55)	*W* = 0.00	< .001
Mean HAM-D Score (SD)	5.59 (6.67)	25.85 (7.26)	*t*_df=35_ = −8.78	< .001

CAPS, Clinician Administered PTSD Scale; HAM-D, Hamilton Rating Scale for Depression.

^a^Insecure asylum status refers to legal situations that imply possible deportation.

^b^ *t*-test for continuous data if test residuals were normally distributed, Mann–Whitney *U*-test for continuous data if residuals were not normally distributed, and Fisher’s Exact test for categorical data.

The study procedures followed the Declaration of Helsinki and were approved by the Ethics Committee of the University of Konstanz. Written informed consent was obtained from the subjects before study participation.

### Clinical interviews

Psychodiagnostic interviews were administered by trained psychologists specialized in the field of trauma, with the assistance of trained interpreters, if required. PTSD diagnosis and the severity of PTSD symptoms were assessed with the *Clinician Administered PTSD Scale* (CAPS) [25]. Furthermore, we calculated separate scores for the three PTSD symptom clusters (intrusions, avoidance and hyperarousal). The number of traumatic event types experienced was ascertained with the respective event list of the CAPS. The *Mini International Neuropsychiatric Interview* (M.I.N.I.) [26] was employed to assess the possible presence of other mental health disorders. Additionally, the severity of depressive symptoms was determined with the *Hamilton Depression Rating Scale* (HAM-D) [27].

### Blood sampling and processing

Peripheral blood was collected by venous puncture into 8.5 ml SST II Plus Vacutainers (BD, USA) before the psychodiagnostic interview (10 am ± 15 min). Participants were asked to have regular breakfast in the morning before the interview to minimize additional strains and prevent circulatory disturbances. The collection containers were directly inverted and stored for 30 min at room temperature to stabilize the blood. Subsequently, serum was separated by centrifugation for 10 min at 2000 g and serum aliquots of 250 μl were immediately stored at −80°C.

### Sample preparation for metabolite profiling

Serum samples were thawed on ice for metabolite extraction. For each participant, a volume of 200 μl serum was taken from the 250 μl aliquot and mixed with 600 μl ice-cold methanol:chloroform (2:1, v/v, Sigma Aldrich, High Pressure Liquid Chromatography (HPLC)-certified). Samples were mixed three times (Vortex Genie 2, Scientific Instruments, USA) for one min, and incubated for 5 min at 4°C in between. The last mixing was performed for 30 sec followed by 10 min incubation on ice. Next, 200 μl ice-cold HPLC-certified water was added and the samples were mixed for 1 min, followed by centrifugation at 14.000 rpm for 5 min at 4°C. Subsequently, the liquid phase of each sample was extracted into a new reaction tube. The samples were vacuum-dried in a CentriVap concentrator linked to a −80°C cold trap (Labconco, USA), sealed (Parafilm, Brand, Germany) and shipped to the National Environmental Research Institute (NERI) at the National University of Singapore (NUS). After arrival samples were re-suspended in 40 μl HPLC-certified water, incubated for 10 min on ice, and centrifuged at 4°C for 15 min at 15.000 rpm. For each sample, a 30 μl supernatant was transferred into HPLC vials (Agilent Incorporated, USA). Samples of subjects with PTSD and controls were analyzed in batches in randomized order.

### Metabolite profiling by liquid chromatography coupled to mass spectrometry

We extracted 5 technical replicates per participant, which were consecutively analyzed by liquid chromatography (LC; model G4226A Agilent Technologies, USA) coupled to a Quadrupole/Time-Of-Flight Mass Spectrometer (QTOF-MS; model G6540A Agilent Technologies, USA). The LC system comprised two Agilent EC-C18 Poroshell columns (2.1 × 5 mm and 2.1 × 100 mm, respectively) with a particle size of 2.7 μm and a pore size of 120 Å. A reference solution contained two references (ions m/z 121.0508 and 922.0097) for continuous autocalibration of QTOF-MS during analyses with a minimum detection threshold of 1000 counts. For metabolite separation, the following LC parameters were set: 2 μl injection volume, 0.3 ml/min binary pump flow, 600 bar high pressure limit, and 4°C autosampler temperature. The mobile phase was composed of two solvents: Solvent A consisted of 0.1% formic acid (FA; Sigma Aldrich) in ultrapure water (TKA Ultrapure Water System) and solvent B consisted of 0.1% FA in acetonitrile (Sigma Aldrich). The gradient cycle started with a solvent composition of 95% of solvent A and 5% of solvent B and reached a solvent composition of 100% solvent B within 9.5 min. The initial solvent composition was re-established prior to the next measurement, resulting in an overall run time of 16 min/sample. The injection needle was washed for 10 sec after each completed run. Mass spectrometric analysis was performed in both positive and negative ionization mode with a scan rate of 2 spectra/sec, a mass range of 100–1700 (m/z), a capillary voltage setting of 4000 V and 3500 V (positive and negative mode, respectively) and a fragmentor setting of 100 V. The pressure of the nebulizer was set to 40 psi, the gas temperature to 250°C, and the continuous gas flow to 12 l/min.

### Raw data processing and quality control

Raw data was imported into the *MassHunter Qualitative Analysis* Software (Agilent Incorporated, USA) for compound feature extraction based on assessed mass and retention time. Features with a minimum absolute abundance of 1000 counts within a defined mass accuracy (<15 ppm) were selected, while no retention time-filtering was applied. The resulting non-normalized data was exported as CEF-files and loaded into *Mass Profiler Professional* software (MPP; Agilent Technology, version 12.5). Feature alignment based on mass (20 ppm + 2.0 mDA tolerance) and retention time (0.5% + 0.15 min tolerance) was performed using the software solution *IDBrowser* (version B.05) implemented in MPP. A recursive analysis considering all possible isoforms of the metabolites was performed in order to enhance accuracy and coverage of metabolite identification. Following this approach, 382 entities were identified in the positive ionization mode and underwent manual filtering based on origin (exogenous vs. endogenous/essential metabolites) and data quality. Initially, all exogenous metabolites were excluded resulting in a number of 138 remaining entities. All metabolites were included that were detected in at least 10 PTSD cases and 10 control subjects. Finally, for each detected metabolite, the median number of replicates per person had to exceed 2 for inclusion. This resulted in a total number of 60 metabolites from the positive ionization mode which entered statistical analysis. The same procedure was repeated for the metabolites detected in the negative ionization mode (N = 178). However, since manual filtering revealed much lower data quality of this assessment (only 6 of 68 endogenous metabolites passed our predefined quality criteria), we decided to restrict the analyses to the 60 entities detected in positive ionization mode. Prior to all statistical analyses, metabolite data was log2 transformed to account for deviances from normal distribution. Data quality control included two steps: First, we manually inspected the consistency of the chromatogram (Additional file 1: Figure S1A). Second, we investigated the results of a principal component analysis (PCA) including all 190 measured probes (5 technical replicates for 38 individuals). The results showed that the 5 technical replicates for each participant clustered together (Additional file 1: Figure S1B). Subsequently, for each metabolite, the average value of the 5 technical replicates of each participant was calculated. Data were then exported from MPP and loaded into the statistical environment R version 3.1.0 [28] for further analyses.

### Statistical analyses

Demographic and clinical variables were compared between PTSD cases and controls using *t*-test or Mann–Whitney-*U*-test if the residuals were not normally distributed for continuous variables and Fisher’s exact test for categorical data. We employed two complementary statistical analyses to identify metabolites associated with PTSD status: 1) univariate analyses to identify group differences in mean abundance values between PTSD cases and controls and 2) *Partial Least Squares Discriminant Analysis* (PLS-DA) as implemented in the R package mixOmics version 5.0-1 [29] as a multivariate approach to identify the best combination of metabolites which separate PTSD cases and controls.

### Dealing with missing data

Several metabolites were not detected in all individuals, which could be due to either a metabolite concentration in this sample below the default signal-to-noise threshold of 1000 counts, or a non-identification of that metabolite due to methodological reasons. For example, as not all ionized metabolites reach the mass spectrometer after nebulization, this results in a variance in the detection rates and availability of data for each replicate. We decided to omit missing data for the univariate analyses. However, since cross-validation is only feasible without missing data, the multivariate analyses were performed after imputing missing data employing the NIPALS algorithm [30] implemented in the R package mixOmics version 5.0-1 [29].

### Univariate statistics

The abundance scores of the single metabolites were analyzed parametrically using Welch’s *t-*tests or non-parametrically using Mann–Whitney-*U*-test, if the residuals of the *t*-test were not normally distributed. The resulting *p*-values were corrected for multiple comparisons (N = 60). Since this was the first exploratory study to investigate metabolite alterations in PTSD we employed the false-discovery rate (FDR) [31] as a correction method for multiple comparisons – an approach that is especially recommended for discovery studies [32,33]. As a compromise between the stringent 5% level and the suggestion of more relaxed FDR thresholds (up to 20%) for an initial discovery study [33], which have been implemented in untargeted metabolite investigations [23], the critical FRD threshold was defined as 10% for this study. All nominal significant metabolites were considered as interesting candidates for further investigations in PTSD and are hence reported, however, only metabolites with a *p*-value < 5% in combination with an FDR < 10% can be considered as associated with PTSD in the narrower sense.

In addition to group comparisons, Kendall’s *τ* correlations were calculated between metabolite signals and PTSD symptom severity (CAPS sum score), PTSD symptom scores (intrusions, avoidance and hyperarousal) as well as with trauma exposure (number of traumatic events assessed in the CAPS). Since there is a high co-morbidity between PTSD and depression in the aftermath of traumatic stress [34], we also tested the association between the identified metabolites and depressive symptomatology (HAM-D).

### Multivariate statistics

While univariate statistics can identify significant group differences in single metabolite levels, multivariate statistics allow for the simultaneous consideration of all investigated metabolites, for the ranking of metabolites according to their importance in predicting PTSD status, and ultimately for the identification of a potential biomarker panel which differentiates PTSD cases from controls. Further, metabolites which do not yield significant univariate group differences can, when combined with other metabolites in a multivariate model, decisively contribute to a clear discrimination between cases and controls [35].

PLS-DA is a multivariate class prediction method especially suited if the number of predictors exceeds the number of observations, which can also deal with multicollinearity among predictors [30,36]. PLS-DA combines the approaches of principal component analyses aiming at dimension reduction and regression analysis. In PLS-DA, all predictor variables X (the metabolites) are projected to a limited number of dimensions termed X-components. The components are extracted in a way that they not only maximize the explained variance in the X-space, but also maximize the covariance between the X-components and the nominal Y-variable [36]. Likewise, metabolites which highly differentiate between cases and controls have a higher importance in defining the components than “noisy” metabolites which are not associated with the disorder status. The relative explanatory power of a metabolite for the resulting class prediction model can be summarized by the *Variable Importance in the Projection* (VIP) factor, which reflects the relevance of a metabolite over all weighted components [36,37]. As the average of the squared VIP equals 1, metabolites with a VIP larger than 1 are generally considered important for the projection. In order to define a biomarker set, metabolites exceeding a certain VIP threshold (usually 1, but higher thresholds may be more accurate in the presence of multicollinearity) can be selected and the PLS-DA model is refitted including only these metabolites [36-38].

We employed 1000 repeats of 10-fold cross-validation to identify the PLS-DA model with the highest predictive accuracy. In order to promote a parsimonious prediction, the one-standard error (SD) rule was applied for model selection [39]. Accordingly, the most economic model whose mean prediction accuracy was < 1 SD below the model with the highest accuracy was chosen.

Employing the above described cross-validation procedure, we first estimated the predictive accuracy of PLS-DA models extracting one to four components. Model accuracy did not enhance with extracting more than three components, and according to the 1 SD rule, a model extracting two components was chosen. In a second step, we compared the models resulting from the extraction of biomarker panels based on six different VIP thresholds (ranging from 1.0 to 1.5). Finally, we evaluated the predictive accuracy of a logistic regression model including only the metabolite with the highest VIP as a predictor.

## Results and discussion

In accordance with our hypothesis, convergent evidence from univariate and multivariate analyses indicates PTSD-associated alterations in the metabolite profile.

### Univariate statistics

**Figure 1:**
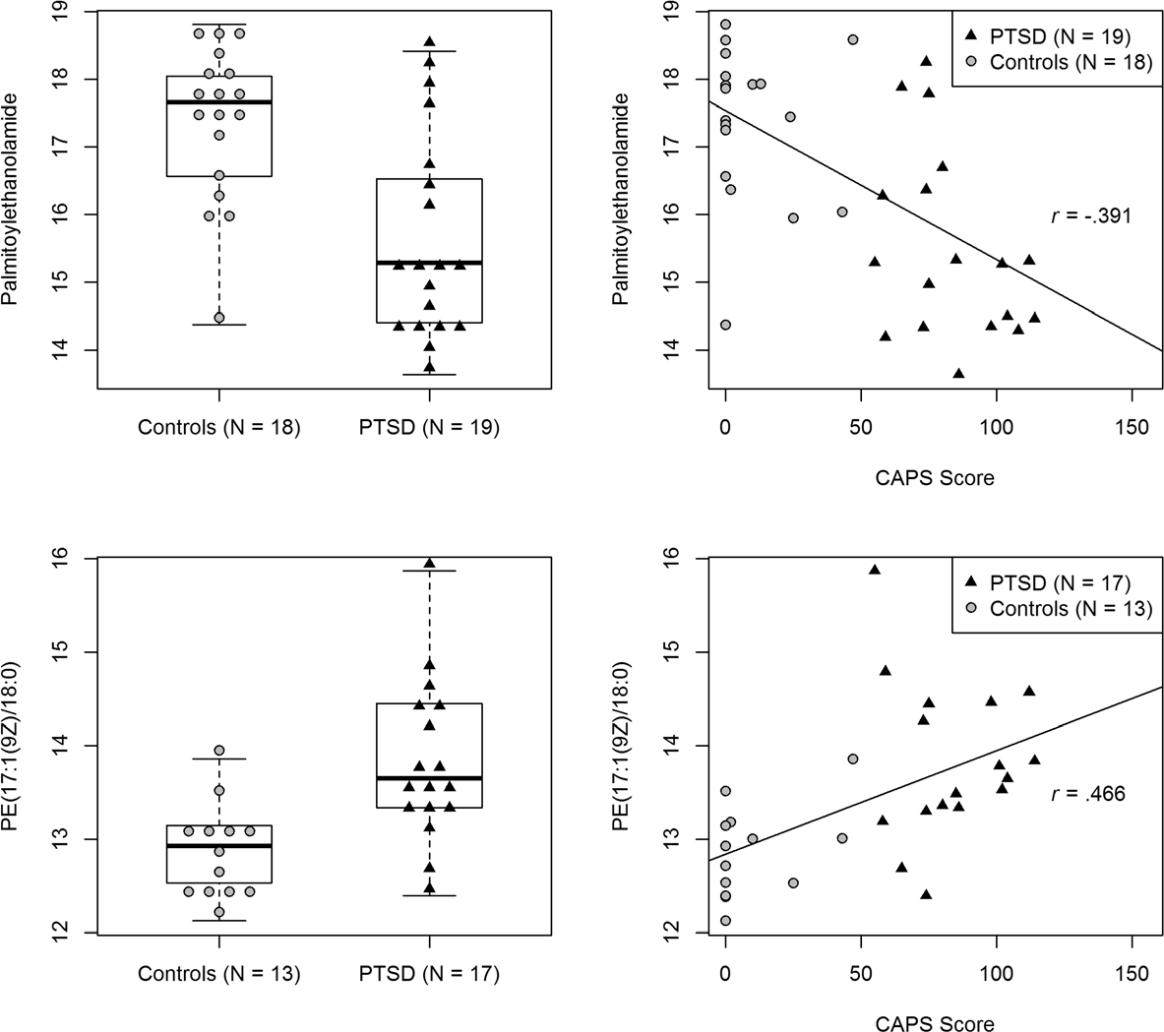
**Displayed are the log2 transformed relative concentrations of Palmitoylethanolamide and the phospholipid PE(17:1(9Z)18:0), which showed the strongest univariate group differences.** The left panel visualizes the differences between PTSD patients and controls, while the right panel displays the correlation with PTSD symptomatology assessed with the Clinician Administered PTSD Scale (CAPS) Score.

Group comparison revealed 13 metabolites, which reached nominal significance (*p* < .05). After multiple comparison correction, two metabolites remained significant at the 5% level (palmitoylethanolamide and PE(17:1(9Z)/18:0); Figure 1) and an additional four metabolites had a FDR of less than 10% (Table 2).

**Table 2:** **Metabolites with significant group differences and their relationship with clinical symptoms**

**Substance class**			**Comparison PTSD vs. Controls**			**Symptom correlation**		
	**Metabolite**	**VIP**	***p***	**FDR**	***d*** **[95% CI]**	***r*** **CAPS**	***p***	**FDR**
*Glycerophospholipids*								
	PE(17:1(9Z)18:0) ↑	1.94	.0007	.020	−1.38 [−2.28, −0.48]	.466	.0004	.019
	PE(P-18:1(11Z)/15:0) ↑	1.70	.004	.073	−1.04 [−1.84, −0.24]	.324	.008	.058
	PE-Nme(O-14:0/O-14:0) ↑	1.46	.007	.073	−0.92 [−1.63, −0.21]	.270	.020	.102
	PE-NMe2(O-14:0/O-14:0) ↑	1.37	.023	.163	−0.79 [−1.51, −0.06]	.185	.125	.325
*Fatty acid metabolites*								
	Palmitoylethanolamide ↓	1.91	.0004	.020	1.28 [0.54, 2.02]	-.391	.001	.019
	Palmitic amide ↓	1.17	.044	.204	0.67 [−0.02, 1.37]	-.190	.102	.294
*Nucleosides*								
	Guanosine ↓	1.22	.039	.204	0.71 [−0.01, 1.43]	-.391	.0009	.019
	Inosine ↓	1.25	.041	.204	0.69 [0.00, 1.39]	-.356	.002	.027
*Bile acids and derivates*								
	3α-hydroxy-5β-cholan-24-oic acid ↓	1.53	.006	.073	0.89 [0.18, 1.6]	-.347	.003	.029
	7α,12α-dihydroxy-3-oxocholest-4-en-26-oic acid ↑	1.55	.007	.073	−0.98 [−1.72, −0.24]	.310	.011	.065
	Glycocholic Acid ↓	1.53	.023	.163	0.82 [0.12, 1.53]	-.301	.016	.085
*Monosaccharides*								
	N-Acetylglucosamine-6-phosphate ↓	1.50	.024	.163	0.83 [0.05, 1.61]	-.392	.002	.027
*Anti-Oxidants*								
	Pantothenic Acid ↓	0.94	.044	.204	0.55 [−0.14, 1.24]	-.323	.005	.047

VIP, Variable Importance in Projection; FDR, False Discovery Rate corrected *p*-values; *d*, Cohen’s *d*; CI, Confidence Interval; CAPS, Clinician Administered PTSD Scale; *r*, Kendall’s *τ* correlations; ↑ and ↓ refer to up- and down-regulation in PTSD, respectively.

NB: The univariate group comparisons PTSD vs. controls were performed by *t*-tests, if test residuals were normally distributed and Mann–Whitney *U*-test if residuals were not normally distributed.

All but two of the identified metabolites also showed strong and significant correlations with the CAPS score (Table 2). Furthermore, for several metabolites (e.g., guanosine, inosine), the relationship with PTSD symptomatology was stronger than the group differences between cases and controls, indicating a dose-dependent relationship between PTSD symptoms and metabolic alterations (Table 2). Similarly, high correlations were found with all three CAPS symptom scores (Additional file 1: Table S1). While correlations between trauma exposure and relative metabolite concentrations generally showed the same direction as CAPS correlations, they were much weaker, and only one metabolite displayed nominal significance (Additional file 1: Table S1), suggesting that PTSD symptoms lead to metabolite alterations beyond the effect of trauma exposure. Similarly, weak correlations were found with depressive symptoms, with only three correlations reaching nominal significance (Additional file 1: Table S1). This finding might be partially explained by the high comorbidity and symptom overlap between PTSD and depression.

### Multivariate statistics

**Figure 2:**
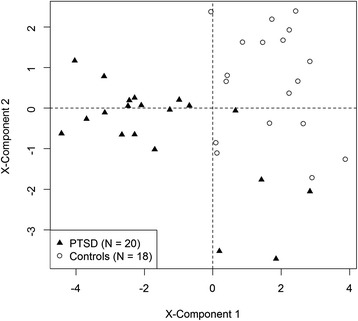
**Separation of PTSD cases and controls in the selected** ***Partial Least Squares Discriminant Analysis*** **(PLS-DA) model including a panel of 19 metabolites.**

A PLS-DA model with two components extracting all metabolites exceeding a VIP threshold of 1.1 yielded the highest predictive accuracy in the cross-validation procedure (see Additional file 1: Figure S2 for a comparison of model accuracy). The selected model included 19 metabolites (Additional file 1: Table S2), of which 12 were also identified by the univariate analyses. In 1000 repeats of 10-fold cross-validation, these 19 metabolites predicted PTSD status with an accuracy of 0.85 (SD = 0.02), a sensitivity of 0.83 (SD = 0.03), and a specificity of 0.87 (SD = 0.03). Figure 2 depicts the separation of PTSD cases and controls on the two components of this PLS-DA model.

We finally investigated the predictive ability of the metabolite with the highest discriminative ability in the PLS-DA model (PE(17:1(9Z)18:0)) by means of cross-validation. A logistic regression model revealed that this metabolite predicted PTSD status with an accuracy of 0.82 (SD = 0.02), rendering this metabolite an interesting biomarker candidate for PTSD. In comparison, the predictive accuracy of the second important metabolite in the PLS-DA model (palmitoylethanolamide) only revealed a predictive accuracy of 0.74 (SD = 0.01).

Multivariate and univariate statistics revealed convergent results, as 12 out of the 13 nominally significant metabolites in the univariate approach were also identified as important predictors of PTSD status by the multivariate approach (Additional file 1: Table S2). Furthermore, both the multivariate and the univariate approach similarly revealed palmitoylethanolamide (PEA) and PE(17:1(9Z)/18:0) as the most important metabolites in the separation of PTSD cases and controls. Accordingly, they were identified as the strongest candidates for an involvement in the pathophysiology in PTSD.

In total, this study identified 20 metabolites potentially associated with PTSD-related psychopathology, which can be subdivided into the following categories: 1) glycerophospholipids, 2) fatty acid metabolites, 3) nucleosides, 4) bile acids, 5) monosaccarides, and 6) anti-oxidants. The more extensive collection of metabolites discovered in the PLS-DA is considered as a useful metabolite panel discriminating PTSD cases from controls. A subset of these metabolites revealed univariate significant group differences, and consequently, can be further discussed regarding the identified up- or down-regulation in PTSD and potential underlying molecular processes involved in disorder psychopathology.

### Glycerophospholipids

Glycerophospholipids are composed of fatty acids and phosphate, and constitute the key components of all cellular membranes. As these barriers not only separate the extracellular milieu from the cytosol, but also contribute to intracellular organization, phospholipids are essential for intracellular metabolism and transmembrane signaling [40].

We identified seven phospholipids which contributed to the differentiation between PTSD cases and controls in the PLS-DA model (Additional file 1: Table S2). Four of these phospholipids, all members of the class of glycerophosphoethanolamines, showed significantly elevated serum concentrations in PTSD. One of these glycerophosphoethanolamines, PE(17:1(9Z)/18:0), had the highest discriminative ability in the multivariate approach. Glycerophosphoethanolamines are catabolites of phophatidylethanolamine, one of the most prevalent mammalian membrane phospholipid, which constitutes approximately 45% of the total phospholipid pool in the brain [41]. Elevated levels of brain glycerophosphoethanolamines have been reported in elderly subjects with depression [42], as well as in Alzheimer’s Disease [43], and may reflect enhanced cell membrane breakdown and inflammation in these subjects [42]. In accordance with these findings, enhanced inflammatory processes have been also reported in PTSD [6,7]. Taken together, the investigation of glycerophosphoethanolamines in the context of PTSD and other stress-related disorders may reveal important insights in the underlying biomolecular processes, which may include (neuro-) inflammation and alterations in cell membrane dynamics and metabolism.

### Fatty acid metabolites

Fatty acids are not only constituents of the aforementioned phospholipids, but are also involved in energy metabolism and serve as signaling molecules. Of the four fatty acid metabolites identified by the PLS-DA approach, PEA is of particular interest due to its involvement in the endocannabinoid system and its potential role in stress-related psychopathology. In our univariate analysis, PEA was down-regulated and showed the strongest association with PTSD status. PEA is a lipid signaling molecule which is formed from membrane phospholipids when required [44]. PEA modulates the endocannabinoid system by potentiating the effects of anandamide, a central agonist of the cannabinoid receptor [45]. In recent years, the interest in PEA has grown due to its anticonvulsant [46], anti-inflammatory and analgesic pharmacological action [47], as well as its neuroprotective effects against oxidative stress [48]. In healthy individuals, PEA serum levels were found to be increased immediately after a social stressor [49] and decreased in the subsequent recovery phase [50]. Similarly, PEA was found to be decreased 24 hours after acute stress in cardiac tissue in rodents [51]. Thus, reduced PEA levels seem to be an important mediator in the link between psychological stress and associated physical health impairments [51]. Furthermore, PEA administration was found to reduce depression and anxiety-like symptoms in animal models of depression [52,53], supporting the hypothesis that PEA may represent a valuable treatment option for depression [54]. So far, the three studies investigating the role of PEA in PTSD found inconsistent results: while Hauer and colleagues [55] reported enhanced PEA levels in PTSD as opposed to trauma-exposed and unexposed controls, two studies did not observe PTSD-associated differences in PEA levels [56,57]. Yet, our results indicate a down-regulation of PEA in PTSD. While future studies are warranted to better understand these inconsistent results, the observed PEA down-regulation in PTSD in this study provides a potential psychobiological explanation for high anxiety and depression symptoms, as well as adverse physical health outcomes observed in this disorder.

In contrast to the large literature on PEA, little is known about palmitic amide (also termed palmitamide), a metabolite belonging to the category of primary fatty acid amides [58], which we found to be significantly down-regulated in PTSD. Due to its structural similarities with PEA, one study investigated the anti-convulsative properties of palmitic amide and found that it also exerts a mild inhibitory effect on seizure frequency in mice [45]. The two remaining metabolites (N-Palmitoyl alanine and 10-Nitrooleate) contributed to the separation in the PLS-DA model, but did not exert significant univariate group differences. Like PEA, both substances have been discussed regarding anti-inflammatory effects [59,60].

### Nucleosides

Nucleosides are constituents of nucleic acids, which are implied in the modulation of several brain processes and psychopathological alterations, including memory, sleep, depression and schizophrenia [61]. The purin-nucleosides guanosine and inosine contributed to the separation of the two groups in the PLS-DA model and were significantly down-regulated in PTSD. These metabolites were found to have neuroprotective properties [62,63] and exert antidepressant-like effects [64,65]. Further, guanosine exerts anxiolytic effects, which might be mediated via its antagonistic effects on glutaminergic signaling [66]. Hence, a down-regulation in PTSD matches well with the psychological and physical symptoms associated with PTSD.

### Bile acids and derivates

Besides their function to transport and absorb nutrients, bile acids are also important signaling molecules for the regulation of lipid, glucose and energy metabolism [67]. We identified four metabolites belonging to the class of bile acids and derivates which contributed to the separation of PTSD cases and controls in the PLS-DA model (Additional file 1: Table S2), of which three also revealed significant group differences in the univariate analysis (Table 2). While the physiological processes linking alterations in bile acids to PTSD etiology remain to be elucidated, one potential hint may be the reported association of bile acid concentration with human aging [68], as accelerated biological aging has been observed in PTSD [9,11].

### Monosaccharides

N-Acetylglucosamine-6-phosphate, a monosaccharide, is a precurser of uridine diphosphate N-acetylglucosamine (UDP-GlcNAc) in the hexosamine pathway. An increase in UDP-GlcNAc was found to inhibit autoimmune reaction and to protect against auto-inflammatory diseases by suppressing T-cell functioning [69,70]. N-Acetylglucosamine-6-phosphate contributed to the group separation in the PLS-DA model and was significantly down-regulated in PTSD. This might contribute to the observed higher rates of auto-immune and inflammatory diseases in PTSD [71].

### Anti-oxidants

We identified two metabolites (4Z,15E-bilirubin IXa and pantothenic acid) with potential anti-oxidant properties. 4Z,15E-bilirubin IXa, an isomer of bilirubin, contributed to the separation of PTSD cases and controls in the PLS-DA model, but did not reach statistical significance in the univariate approach. Modestly elevated levels of bilirubin exert protective effects against oxidative stress, inflammation and atherosclerotic disease, yet high levels of circulating bilirubin are often cytotoxic [72]. By contrast, pantothenic acid (also termed vitamin B5) was found to be significantly down-regulated in PTSD, but did not potently contribute to the separation between PTSD cases and controls and hence was not included in the PLS-DA model. This vitamin is a precursor of coenzyme A, exerts antioxidant action [73] and might therefore exert a protective function against the mitochondrial oxidative decay of aging [74].

### Strengths, limitations, and future research directions

Strengths of the study include the comprehensive diagnostic interviews, and the assessment of individuals with varying degrees of trauma exposure, which allowed us to investigate potential dose-dependent effects of trauma exposure and PTSD symptomatology. The major limitation of this first study on metabolite alterations in PTSD is the relatively small sample size and the lack of an independent replication sample. Therefore, differences between the diagnostic groups apart from the PTSD diagnosis could have also contributed to the observed metabolite alterations. One potential confounding factor might be recent dietary influences, as the study participants were asked to have breakfast prior to the examination. This decision was made in order to avoid additional stress and potential circulatory disturbances for the trauma survivors, who often travel longer than an hour from the asylum seeker accommodation to the University of Konstanz. Furthermore, it is frequently discussed that PTSD is associated with engagement in health risk behaviors including smoking, alcohol or drug abuse. However, we excluded individuals who met criteria of substance addiction, and the two diagnostic groups did not differ in smoking behavior (cf. Table 1). Additionally, the entire PTSD group opposed to seven individuals in the control group faced an unsecure asylum status. Hence, one might argue that the strain associated with a possible deportation could account for some of our findings. While this assumption cannot be completely eliminated given our small sample size, at least two arguments are in contrast with this explanation. First of all, the observed dose–response relationship between PTSD symptom severity and alterations at the metabolite level (cf. Figure 1, Table 2) contradicts the idea that our findings might be merely accounted by the asylum status. Second, we exploratorily investigated for PEA and PE(17:1(9Z)/18:0) if individuals from the control group with an insecure asylum status clustered together and showed metabolite alterations comparable to the PTSD group, which was not the case (compare Additional file 1: Figure S3).

Future targeted and untargeted studies on metabolite alterations in PTSD are warranted to confirm the identified relations. Forthcoming studies should further compare metabolite alterations in PTSD with other stress-related disorders such as depression to identify shared and distinct biological pathways underlying psychiatric diagnoses.

Finally, it would be interesting to investigate in the future whether psychotherapeutic or psychopharmacological treatments for PTSD are accompanied by changes in relative metabolite concentrations and if responders and non-responders could be differentiated *ex ante* based on their metabolite profile.

## Conclusions

In conclusion, metabolites which were found to be associated with PTSD status are involved in processes of (neuro-) inflammation, auto-immune reactions, oxidative stress, energy metabolism, and biological aging. Accordingly, these metabolites provide putative links between the development of PTSD and a higher risk for adverse physical health consequences. The majority of the identified metabolites belonged to the class of phospholipids, and class prediction from one single phospholipid already yielded a good separation between cases and controls, indicating a significant role of lipid-derived signaling in PTSD. Furthermore, the high association between relative PEA concentrations and PTSD supports a contribution of the endocannabinoid system in PTSD etiology.

## Additional file

Additional file 1: Figure S1A. The chromatograms of all 190 measured probes (38 participants × 5 technical replicates) were superimposed to manually inspect the consistency of the characteristic metabolite peaks. **Figure S1B.** The latent structure of all measured probes was investigated by means of a principal component analysis (PCA). The results showed that the five technical replicates per participant clustered together. **Table S1.** Correlation of relative metabolite concentrations with PTSD symptom clusters and trauma exposure. **Figure S2.** The critical Variable Importance in the Projection (VIP) threshold for metabolites to be included in the Partial Least Square Discriminant Analysis (PLS-DA) model was determined by comparing the predictive accuracy of the resulting PLS-DA models by means of 1000 repeats of 10-fold cross-validation. Displayed are the mean predictive accuracies along with the standard deviation for each model. The highest predictive accuracy was reached for a model with a VIP of 1.1. **Table S2.** Metabolites exceeding a VIP of 1.1 included in the Partial Least Squares Discriminant Analysis (PLS-DA) model. **Figure S3.** Displayed are the log2 transformed relative concentrations of palmitoylethanolamide and the phospholipid PE(17:1(9Z)18:0) by diagnostic group. All individuals in the PTSD group, but only seven individuals in the control group faced an insecure asylum status. In order to examine the potential influence of the insecure asylum status on the metabolite levels, we exploratorily inspected the metabolite data of individuals with an insecure asylum status in the control group (displayed as red circles).

## Abbrevations

am: Ante meridiem
min: Minute
g: Gravity
μl: Microliters
C: Celsius
v/v: Percent concentration volume/volume
sec: Second
rpm: Rounds per minute (centrifugation unit)
mm: Millimeter
ml: Milliliter
μl: Microliter
psi: Pound-force per square inch
μm: Micrometer
Å: Angström
mDa: Milli-Dalton
m/z: Mass-to-charge ratio
V: Volt
l: Liter
%: Percent
ppm: Parts per million
