# Supplementary material for: Metabolite profiling in posttraumatic stress disorder
Source: J Mol Psychiatry. 2015 Feb 8;3(1):2. doi: 10.1186/s40303-015-0007-3 (PMC4367823; doi:10.1186/s40303-015-0007-3)
Supplement: Additional file 1: Figure S1A. — The chromatograms of all 190 measured probes (38 participants × 5 technical replicates) were superimposed to manually inspect the consistency of the characteristic metabolite peaks. Figure S1B. The latent structure of all measured probes was investigated by means of a principal component analysis (PCA). The results showed that the five technical replicates per participant clustered together. Table S1. Correlation of relative metabolite concentrations with PTSD symptom clusters and trauma exposure. Figure S2. The critical Variable Importance in the Projection (VIP) threshold for metabolites to be included in the Partial Least Square Discriminant Analysis (PLS-DA) model was determined by comparing the predictive accuracy of the resulting PLS-DA models by means of 1000 repeats of 10-fold cross-validation. Displayed are the mean predictive accuracies along with the standard deviation for each model. The highest predictive accuracy was reached for a model with a VIP of 1.1. Table S2. Metabolites exceeding a VIP of 1.1 included in the Partial Least Squares Discriminant Analysis (PLS-DA) model. Figure S3. Displayed are the log2 transformed relative concentrations of palmitoylethanolamide and the phospholipid PE(17:1(9Z)18:0) by diagnostic group. All individuals in the PTSD group, but only seven individuals in the control group faced an insecure asylum status. In order to examine the potential influence of the insecure asylum status on the metabolite levels, we exploratorily inspected the metabolite data of individuals with an insecure asylum status in the control group (displayed as red circles). [file 40303_2015_7_MOESM1_ESM.docx]

**Supplementary Information**

**Metabolite profiling in posttraumatic stress disorder**

Alexander Karabatsiakis^1†^, Gilava Hamuni^1†^, Sarah Wilker^1†^, Stephan Kolassa^2^, Durairaj Renu^3^, Suzanne Kadereit^4^, Maggie Schauer^5^, Thomas Hennessy^6^ & Iris-Tatjana Kolassa^1^

† These authors contributed equally to this article

^1^ Clinical & Biological Psychology, Institute of Psychology and Education, University of Ulm, Germany

^2^ SAP Switzerland AG, Tägerwilen, Switzerland

^3^ Strand Life Sciences Pvt. Ltd., Bangalore, India

^4^ Department of Biology, University of Konstanz, Germany

^5^ Clinical Psychology & Neuropsychology, University of Konstanz, Germany

^6^ Agilent Incorporated, Singapore, Republic of Singapore

**Data quality control**


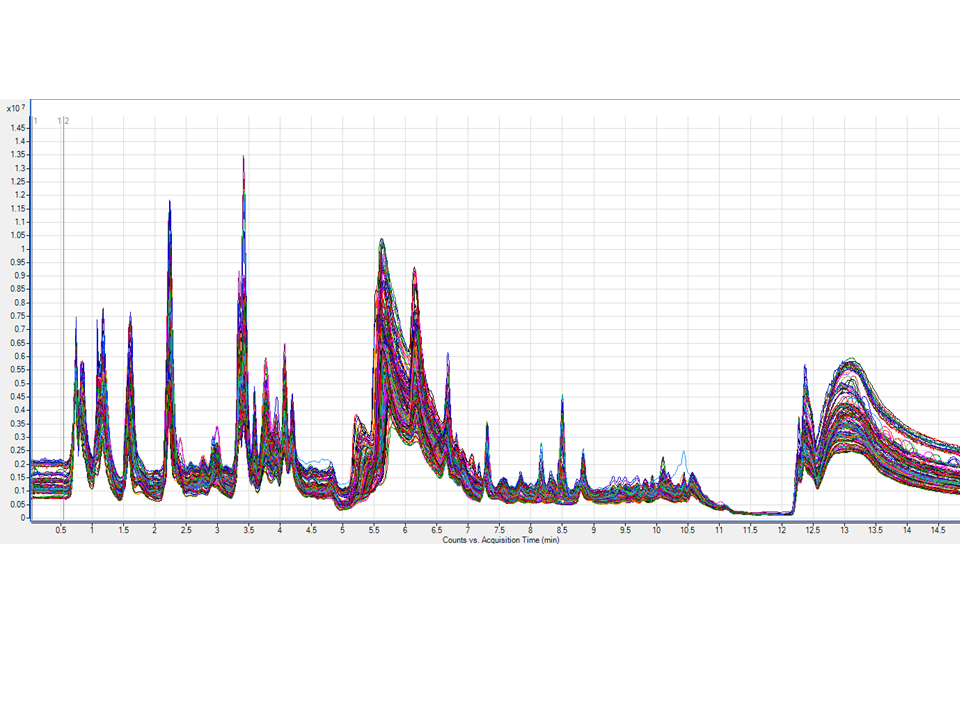


**Supplementary Figure S1A:** The chromatograms of all 190 measured probes (38 participants × 5 technical replicates) were superimposed to manually inspect the consistency of the characteristic metabolite peaks.


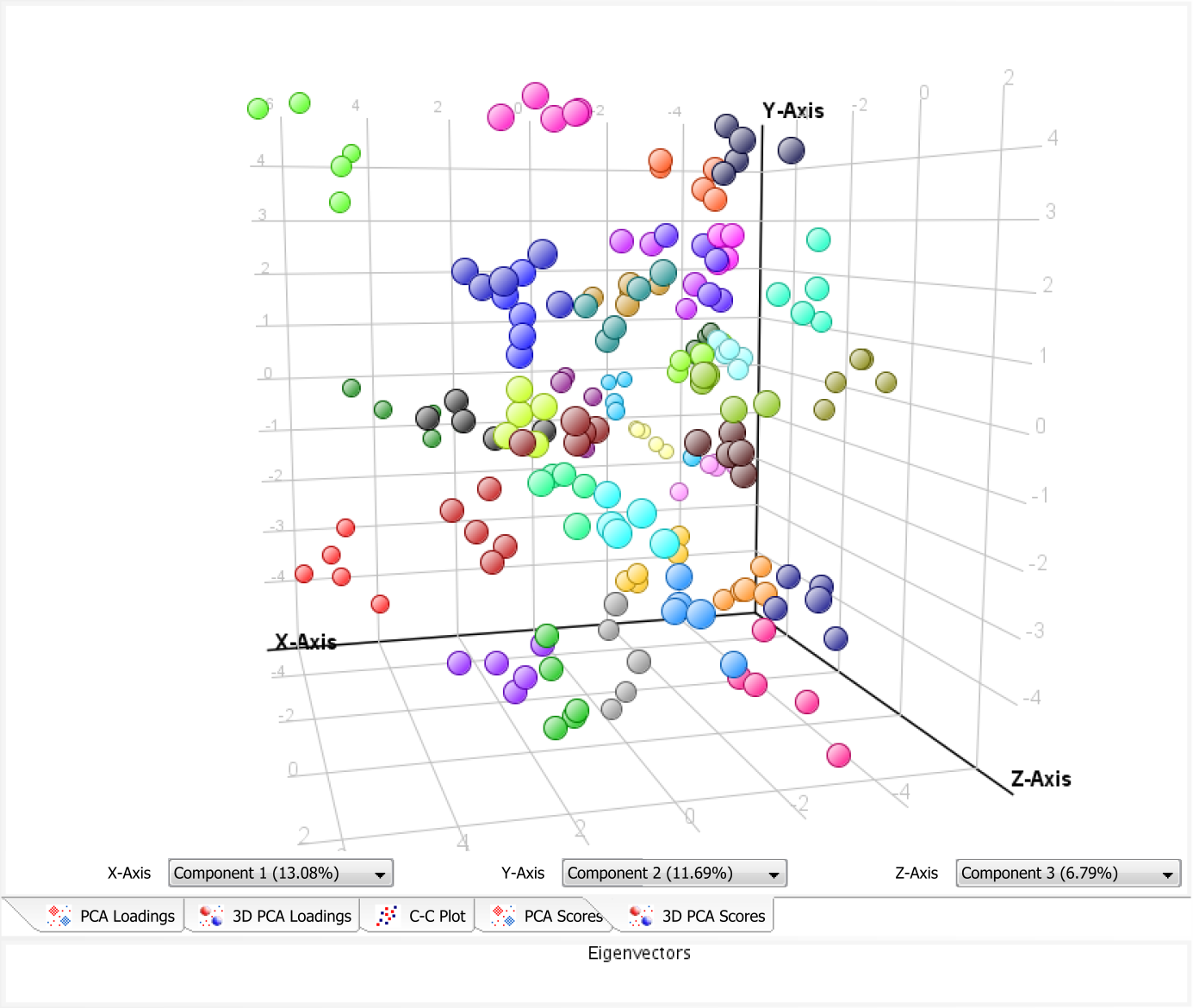


**Supplementary Figure S1B:** The latent structure of all measured probes was investigated by means of a principal component analysis (PCA). The results showed that the five technical replicates per participant clustered together.

**Results**

*Univariate Statistics*

*Supplementary Table S1: Correlation of relative metabolite concentrations with PTSD symptom clusters and trauma exposure*

| **Substance Class** | | | |  | **PTSD Symptom**  **Subscores** | | | |  | **Trauma Exposure** |  | **Depressive Symptoms** | | |
| --- | --- | --- | --- | --- | --- | --- | --- | --- | --- | --- | --- | --- | --- | --- |
|  | | | **Metabolite** |  | ***r* CAPS Intrusions** | ***r* CAPS Avoidance** | ***r* CAPS Hyperarousal** | |  | ***r* CAPS Events** |  | ***r***  **HAM-D** | | |
| Glycerophospholipids | | | |  |  |  | |  |  |  |  | |  |  |
|  | PE(17:1(9Z)18:0) ↑ | | |  | .415** | .462*** | | .426** |  | .337* |  | |  | .317* |
|  | PE(P-18:1(11Z)/15:0) ↑ | | |  | .318* | .318* | | .316* |  | .211 |  | |  | .169 |
|  | PE-Nme(O-14:0/O-14:0) ↑ | | |  | .274* | .258* | | .276* |  | .118 |  | |  | .178 |
|  | PE-NMe2(O-14:0/O-14:0) ↑ | | |  | .198 | .215 | | .150 |  | .044 |  | |  | .106 |
| Fatty acid metabolites | | | |  |  |  | |  |  |  |  | |  |  |
|  | Palmitoylethanolamide ↓ | | |  | -.350** | -.421*** | | -.380** |  | -.137 |  | |  | -.300* |
|  | Palmitic amide ↓ | | |  | -.217 | -.224 | | -.126 |  | -.085 |  | |  | -.215 |
| Nucleosides | | | |  |  |  | |  |  |  |  | |  |  |
|  | Guanosine ↓ | | |  | -.354** | -.396*** | | -.340** |  | -.127 |  | |  | -.160 |
|  | Inosine ↓ | | |  | -.328** | -.369** | | -.273* |  | -.178 |  | |  | -.160 |
| Bile acids and derivates | | | |  |  |  | |  |  |  |  | |  |  |
|  | 3α-hydroxy-5β-cholan-24-oic acid ↓ | | |  | -.349** | -.369** | | -.291* |  | -.072 |  | |  | -.193 |
|  | 7α,12α-dihydroxy-3-oxocholest-4-en-26-oic acid ↑ | | |  | .340** | .269* | | .289* |  | .093 |  | |  | .146 |
|  | Glycocholic Acid ↓ | | |  | -.217 | -.310* | | -.320* |  | -.171 |  | |  | -.144 |
| Monosaccharides | | | |  |  |  | |  |  |  |  | |  |  |
|  | N-Acetylglucosamine-6-phosphate ↓ | | |  | -.352** | -.378** | | -.355** |  | -.019 |  | |  | -.236 |
| Anti-Oxidants | | | |  |  |  | |  |  |  |  | |  |  |
|  | | Pantothenic Acid ↓ | |  | -.247* | -.350** | | -.240* |  | .000 |  | |  | -.264* |

Reported correlations were calculated with Kendall’s τ

CAPS, Clinician Administered PTSD Scale; HAM-D; Hamilton Rating Scale for Depression; stars donate nominal significant thresholds, with * *p* < .05, ** *p* < .01, *** *p* < .001

*Multivariate Statistics*


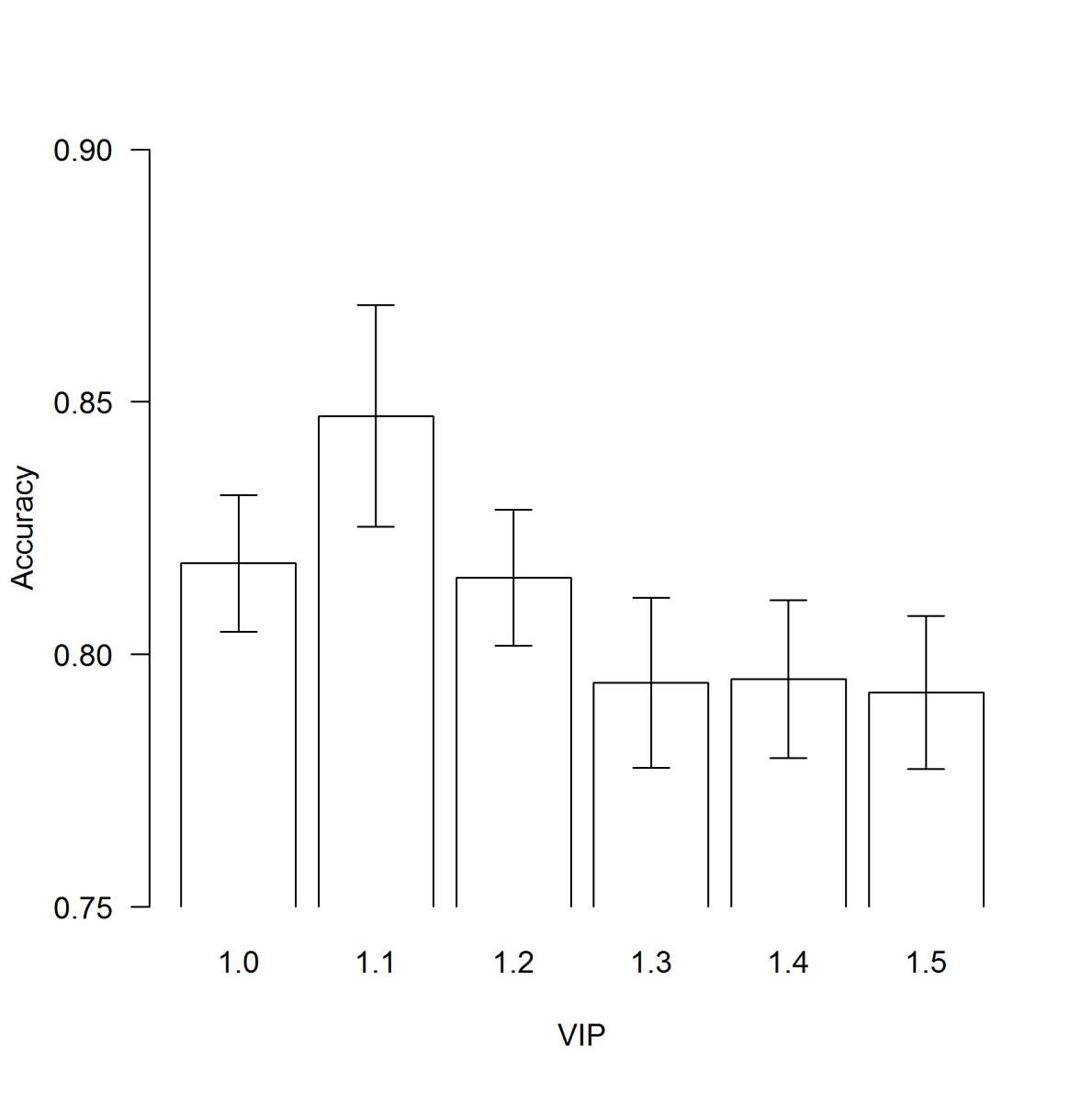


**Supplementary Figure S2:** The critical *Variable Importance in the Projection* (VIP) threshold for metabolites to be included in the *Partial Least Square Discriminant Analysis* (PLS-DA) model was determined by comparing the predictive accuracy of the resulting PLS-DA models by means of 1000 repeats of 10-fold cross-validation. Displayed are the mean predictive accuracies along with the standard deviation for each model. The highest predictive accuracy was reached for a model with a VIP of 1.1.

| **Substance Class** | **Metabolite** | **VIP** |
| --- | --- | --- |
| Glycerophospholipids | PE(17:1(9Z)18:0) | 1.94 |
| Glycerophospholipids | PE(P-18:1(11Z)/15:0) | 1.70 |
| Glycerophospholipids | PE-Nme(O-14:0/O-14:0) | 1.46 |
| Glycerophospholipids | PE-NMe2(O-14:0/O-14:0) | 1.37 |
| Glycerophospholipids | PE(P-18:0/17:1(9Z)) | 1.12 |
| Glycerophospholipids | LysoPE(0:0/18:2(9Z,12Z)) | 1.26 |
| Glycerophospholipids | PG(20:5(5Z,8Z,11Z,14Z,17Z)/0:0) | 1.17 |
| Fatty acid metabolites | Palmitoylethanolamide | 1.91 |
| Fatty acid metabolites | Palmitic amide | 1.17 |
| Fatty acid metabolites | N-palmitoyl alanine | 1.14 |
| Fatty acid metabolites | 10-Nitrooleate | 1.27 |
| Nucleosides | Guanosine | 1.22 |
| Nucleosides | Inosine | 1.25 |
| Bile acids and derivates | 3α-hydroxy-5β-cholan-24-oic acid | 1.53 |
| Bile acids and derivates | 7α,12α-dihydroxy-3-oxocholest-4-en-26-oic acid | 1.55 |
| Bile acids and derivates | Glycocholic Acid | 1.53 |
| Bile acids and derivates | 3α,7α,12α-trihydroxy-6-oxo-5β-cholan-24-oic acid | 1.18 |
| Monosaccharides | N-Acetylglucosamine-6-phosphate | 1.50 |
| Anti-Oxidants | 4Z,15E-Bilirubin IXa | 1.23 |

*Supplementary Table S2: Metabolites exceeding a VIP of 1.1 included in the Partial Least Squares Discriminant Analysis (PLS-DA) model*

VIP, Variable Importance in the Projection. Displayed are the 19 metabolites which contributed to the differentiation between PTSD cases and controls in the PLS-DA model. Twelve of these metabolites also reached nominal significance in the univariate group comparison and are shaded in grey. The corresponding *p*-values and FDR-corrected *p*-values can be found in table 2 of the main text.


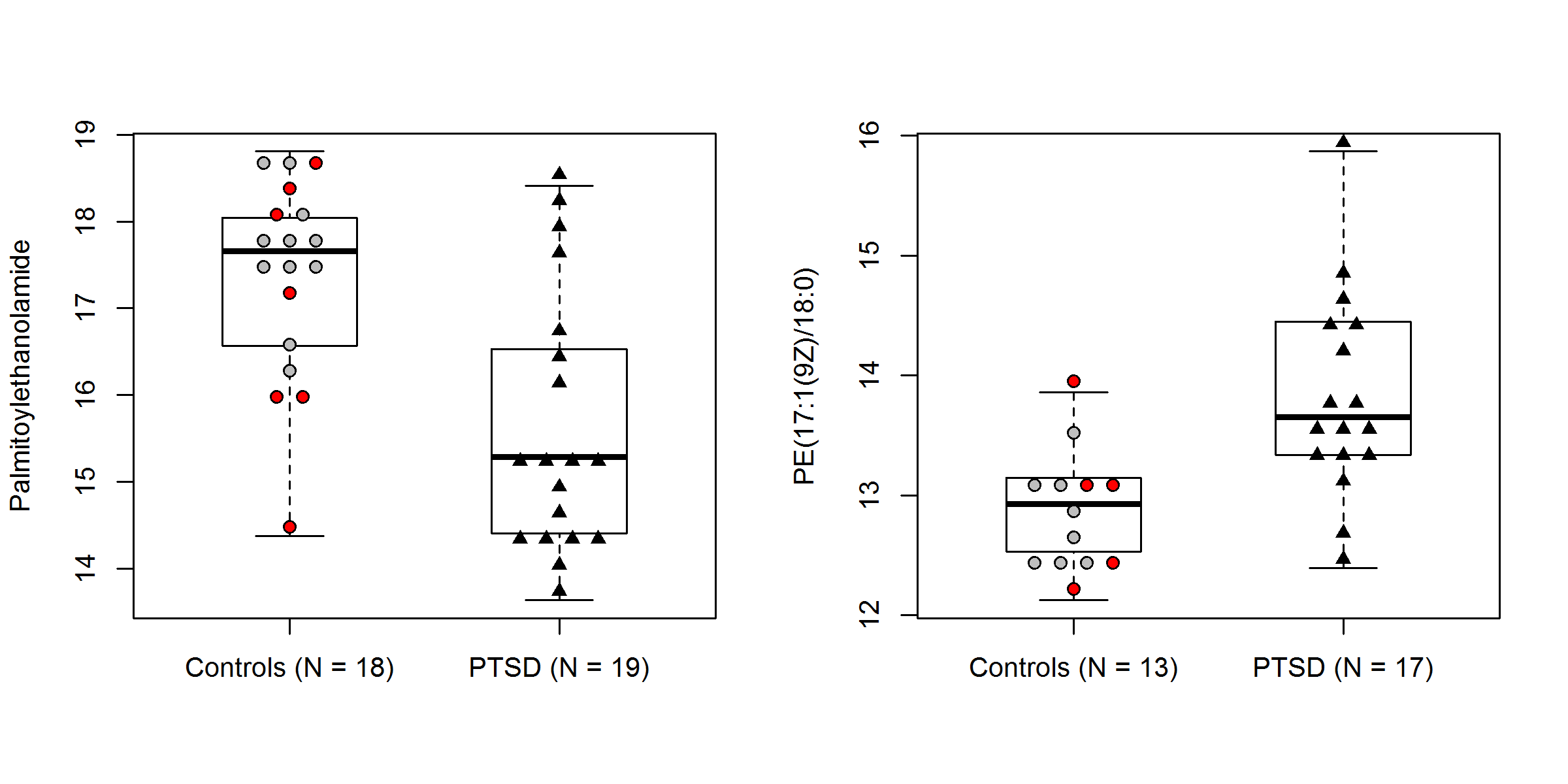


**Supplementary Figure S3:** Displayed are the log2 transformed relative concentrations of Palmitoylethanolamide and the phospholipid PE(17:1(9Z)18:0) by diagnostic group. All individuals in the PTSD group, but only seven individuals in the control group faced an insecure asylum status. In order to examine the potential influence of the insecure asylum status on the metabolite levels, we exploratory inspected the metabolite data of individuals with an insecure asylum status in the control group (displayed as red circles).
